# Time to initial glycopeptide therapy and 30-day mortality in methicillin-resistant *Staphylococcus aureus* bacteremia: a retrospective cohort study

**DOI:** 10.1186/s12879-025-12040-9

**Published:** 2025-11-19

**Authors:** Tae-Hoon No, Seok Jun Mun

**Affiliations:** 1 Division of Infectious Diseases, Department of Internal Medicine, Inje University Haeundae Paik Hospital, Busan, Republic of Korea; 2 Division of Infectious Diseases, Department of Internal Medicine, Inje University Busan Paik Hospital, 75 Bokji-Ro, Busanjin-Gu, Busan, 47392 Republic of Korea; 3 Paik Institute for Clinical Research, Inje University College of Medicine, Busan, Republic of Korea

**Keywords:** Methicillin-resistant *staphylococcus aureus*, Bacteremia, Glycopeptides, Treatment outcome, Time factors

## Abstract

**Background:**

The optimal time cut-off for appropriate antibiotic therapy (AAT) in methicillin-resistant *Staphylococcus aureus* (MRSA) bacteremia remains uncertain. We assessed the effects of time to AAT on 30-day in-hospital mortality in patients with MRSA bacteremia who received glycopeptides as initial therapy, and applied landmark analyses to adjust for immortal-time bias.

**Methods:**

We conducted a retrospective cohort study of adults with MRSA bacteremia who received an initial course of glycopeptide therapy at two university-affiliated hospitals between 2018 and 2023. Multivariable logistic regression was used to identify covariates. These covariates were then included in six separate landmark models that assessed the effect of AAT within pre-specified cut-off time of 3, 6, 12, 24, 48, and 72 h after the index blood culture collection.

**Results:**

Among 220 patients, osteoarticular infections were the focus of bacteremia in 25.5%, and septic shock occurred as a complication in 28.2%; 30-day in-hospital mortality was 24.1%. Vancomycin was administered as initial therapy in 63.2% of patients, and the median time to glycopeptide initiation was 36 h (interquartile range, 19–67.5). Metastatic solid tumor, initial septic shock, pneumonia, and unknown focus were independent risk factors for death according to multivariable analysis. After covariate adjustment, glycopeptide therapy administered within any of the six pre-defined cut-off time was not significantly associated with mortality.

**Conclusions:**

No significant time cut-off for glycopeptide initiation was associated with increased mortality. In patients without septic shock or other mortality-related risk factors, a modest delay in AAT may have slight effect on outcomes.

**Supplementary Information:**

The online version contains supplementary material available at 10.1186/s12879-025-12040-9.

## Introduction

*Staphylococcus aureus* bacteremia (SAB) remains a life-threatening infection, with a reported mortality rate of more than 20%, despite current therapeutic advancements [1]. In particular, bacteremia caused by methicillin-resistant *S. aureus* (MRSA) is challenging owing to limited therapeutic options and consistently poor prognosis [1, 2].

Among the therapeutic factors that influence the prognosis of MRSA bacteremia, the harmful effects of delayed appropriate antibiotic therapy (AAT) have been well documented. However, evidence defining an optimal therapeutic window remains inconclusive [3]. Previous studies have reported AAT cut-off time, ranging from 24 to 48 h, beyond which mortality increases, and some studies have even shown no survival benefit associated with early therapy [4–14]. These diverse findings across SAB studies on the effects of early AAT may stem from methodological concerns that can influence time-to-AAT analyses, such as immortal-time bias and inadequate confounding adjustment [15, 16]. Uncertainty regarding the optimal AAT cut-off time in MRSA bacteremia not only increases the risk of adverse outcomes due to delayed therapy but also promotes the overuse of broad-spectrum antibiotics, thereby fostering antibiotic resistance. Accordingly, after adjusting for potential confounders and correcting for immortal-time bias, we sought to determine the optimal time to AAT in patients with MRSA bacteremia who received glycopeptides as their initial treatment.

## Methods

### Study design and population

We conducted a retrospective cohort study of patients aged ≥18 years with MRSA bacteremia between January 2018 and December 2023 at two university-affiliated hospitals: Inje University Busan Paik Hospital (BPH, 810 beds) and Inje University Haeundae Paik Hospital (HPH, 866 beds). We included patients who had signs or symptoms of infection (fever ≥38 °C, chills, or hypotension) and who either received at least one dose of glycopeptide therapy as initial treatment or died before receiving any anti-MRSA therapy. Exclusion criteria were patients with polymicrobial bacteremia, who were lost to follow-up within 14 days of their initial positive blood culture, who received anti-MRSA therapy before blood culture collection, or whose first positive blood culture was obtained at a different hospital. This study was approved by the Inje University Busan Paik Hospital Institutional Review Board (IRB) and the Inje University Haeundae Paik Hospital IRB, with a waiver of informed consent (IRB numbers: BPIRB 2025–01-013 and HPIRB 2025–01-005).

## Definitions and outcome

Glycopeptide therapy was defined as the administration of intravenous vancomycin or teicoplanin. Metastatic infection was defined as the presence of infection sites that were anatomically distinct from the primary source of bacteremia [17]. Community-onset bacteremia was defined as a positive blood culture obtained from patients within 48 h of hospitalization. Initial septic shock was defined as the administration of vasopressors to maintain a mean arterial pressure ≥ 65 mmHg on the day of the first positive blood culture [11, 18]. The Pitt bacteremia score (PBS) was used to assess acute severity of illness and is composed of five criteria: fever, hypotension, mechanical ventilation, cardiac arrest, and mental status [19, 20]. The duration of bacteremia was calculated from the day of the first positive blood culture to the day of the last positive follow-up blood culture. An eradicable focus was defined as an infection amenable to surgical removal or associated with an indwelling foreign body or drainable abscess [17]. The interval to the initiation of glycopeptide therapy was determined from the time of blood culture collection. The primary outcome measure was 30-day in-hospital mortality. Favorable outcomes were defined as survival beyond 30 days after the first positive blood culture in the hospital or discharge from the hospital while still alive.

## Blood culture and antibiotic susceptibility testing

Blood cultures were obtained from either peripheral veins or central venous catheters. A Bactec-9240 system (Becton Dickinson, Sparks, MD, USA) or BacT/Alert 3D system (bioMérieux Inc., Marcy l’Etoile, France) was used for blood cultures. A VITEK II automated system (bioMérieux, Inc.) was used for antibiotic susceptibility testing.

## Vancomycin therapeutic drug monitoring (TDM)

Vancomycin TDM was performed at the discretion of the treating physicians. During the study period, both hospitals initially employed trough concentration–based dosing, and subsequently transitioned to area under the curve (AUC)–based dosing (BPH, August 2020; HPH, July 2022).

## Statistical analysis

Continuous variables are reported as medians with interquartile ranges (IQR), and categorical variables as frequencies with percentages. After assessing normality, continuous variables were compared using the Mann–Whitney *U* test. Fisher’s exact test or chi-square test was used to compare categorical variables. Univariable logistic regression was performed to assess association with 30-day in-hospital mortality. Covariates showing *p* < 0.10, along with those considered clinically important, were advanced to multivariable analysis. A multivariable logistic regression model was subsequently constructed using forward conditional selection to identify independent predictors. Multicollinearity was examined using variance inflation factors, and a value of > 10 was interpreted as excessive collinearity [21]. Calibration was assessed using the Hosmer–Lemeshow goodness-of-fit test [22], and discrimination was evaluated by the area under the receiver operating characteristic curve (AUROC) [23].

To evaluate the effect of time to initial glycopeptide therapy while limiting immortal-time bias, landmark analyses were conducted at predefined cut-off points of 3, 6, 12, 24, 48, and 72 h from the index blood culture [15]. At each landmark, patients who died before the cut-off were excluded, and a separate multivariable logistic model, adjusted for the covariates retained in the main model, was fitted to estimate the association between the initiation of glycopeptide therapy and 30-day mortality. Since the time to initial glycopeptide therapy of > 120 h after the index blood culture is unusual in clinical practice, we performed a sensitivity analysis in which these patients were excluded and landmark analyses were repeated [16]. Results are presented as adjusted odds ratios (aORs) with 95% confidence intervals (CIs). All statistical tests were two-sided, and values of *p* < 0.05 were considered significant. Statistical analyses were performed using the IBM SPSS Statistics for Windows (version 25.0; IBM Corp., Armonk, NY, USA) and R (version 4.5.0; R Foundation for Statistical Computing, Vienna, Austria).

## Results

### Study population and patient characteristics

**Fig. 1:**
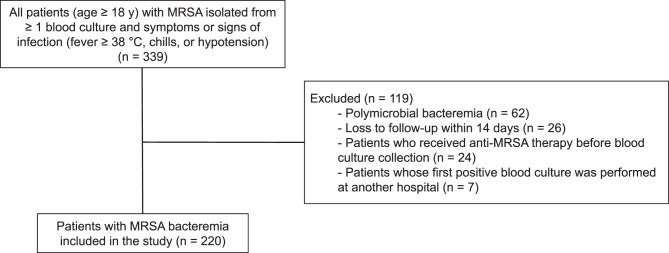
Patient population. MRSA, methicillin-resistant *Staphylococcus aureus*

Of 339 patients with MRSA bacteremia, 220 were included in this study: 122 from BPH and 98 from HPH (Fig. 1). In the overall cohort, 116 patients (52.7%) were male; the median age was 71 years (IQR 61–80), and the median Charlson Comorbidity Index (CCI) was 2 (IQR 1–4.75). The median duration of bacteremia was 1 d (IQR 1–4.75), and 56.8% of episodes were community-onset. Septic shock was present at the onset of bacteremia in 28.2% of patients, and the median PBS was 1 (IQR 0–3). The most frequent foci of infection were osteoarticular sites (25.5%), followed by an unknown focus (20.5%).

Initial vancomycin therapy was administered to 139 patients (63.2%), whereas 14 (6.3%) died before the initiation of glycopeptide therapy. Among patients who received vancomycin, the highest median trough concentration within the first seven days was 15.2 μg/mL (IQR 10.1–19.8). Within the first seven days, the highest trough concentration was ≥15 μg/mL in 67 patients (51.5%), and ≥10 μg/mL in 99 patients (76.2%). Among teicoplanin-treated patients, the median maintenance dose was 6 mg/kg (IQR 5.5–6.3). A loading dose was omitted in five cases; among the remaining recipients, the median loading dose was 6 mg/kg (IQR 5.5–6.7). The median interval from blood culture collection to the initiation of glycopeptide therapy was 36 h (IQR 19–67.5). This interval was significantly longer among patients who received initial vancomycin therapy (median, 44 h; IQR, 23–73) compared with those who received initial teicoplanin therapy (median, 25 h; IQR, 9–54; *p* = 0.002).

**Fig. 2:**
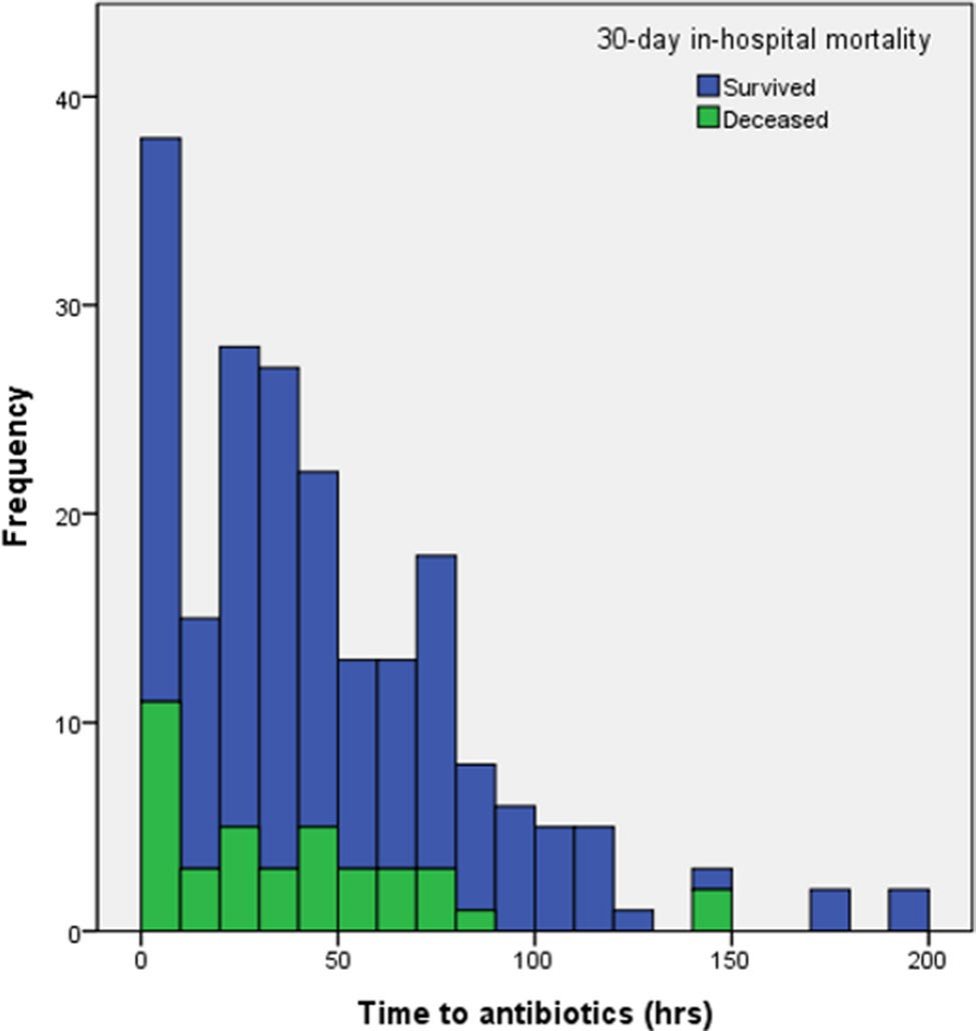
Distribution of time from index blood culture to initial glycopeptide therapy in patients with MRSA bacteremia. MRSA, methicillin-resistant *S. aureus*

Thirtyday inhospital mortality was 24.1%. The median interval from the day of the first positive blood culture to death was 9 d (IQR 3.5–16.5). Forty-two patients survived but were either discharged or lost to follow-up within 30 days; their median follow-up duration was 23 d (IQR 19–27). Compared with survivors, nonsurvivors were older, had a lower CCI, and had frequent metastatic solid tumors (Table 1). Moreover, initial septic shock, higher PBS, pneumonia, and unknown focus were more common among nonsurvivors, whereas osteoarticular infection and removal of the eradicable focus within three days were more frequent among survivors. Vancomycin was administered more often than teicoplanin to survivors, and the interval from bloodculture collection to the initiation of glycopeptide therapy did not differ significantly between the two groups (Table 2, Fig. 2). The median duration of initial glycopeptide therapy was 17 d (IQR 12–29) in survivors and 5 d (IQR 3–13) in non-survivors. Compared with patients at HPH, those at BPH more frequently had nosocomial-onset bacteremia and an unknown focus, and were less likely to undergo removal of the focus after three days (Supplementary Table 1, Additional File 1). Initial teicoplanin therapy was more common among BPH patients, and the overall duration of glycopeptide therapy was longer, whereas the time to initiation of glycopeptide therapy was shorter (Supplementary Table 2, Additional File 1). The 30-day in-hospital mortality did not differ significantly between the two hospitals (23.8% vs. 24.5%).

**Table 1:** Baseline characteristics of patients with methicillin-resistant *S. aureus* bacteremia

Characteristics	Survived (*n* = 167)	Deceased (*n* = 53)	*P* value
Male	87 (52.1)	29 (54.7)	0.739
Age in years, median (IQR)	70 (59–79)	77 (67.0–81.5)	0.002
Comorbidities			
Charlson comorbidity index, median (IQR)	2 (1–4)	3 (1.0–5.5)	0.151
Moderate to severe liver disease	5 (3.0)	5 (9.4)	0.063
Moderate to severe CKD	32 (19.2)	7 (13.2)	0.323
Serum creatinine (mg/dL), median (IQR)	1.06 (0.70–1.81)	1.34 (0.75–2.33)	0.165
Hemodialysis dependence	23 (13.8)	3 (5.7)	0.111
Metastatic solid tumor	15 (9.0)	11 (20.8)	0.021
Prostheses			
Orthopedic device	40 (24.0)	7 (13.2)	0.096
Cardiovascular device	9 (5.4)	2 (3.8)	> 0.999
Long-term CVC	29 (17.4)	10 (18.9)	0.803
Other prosthesis	11 (6.6)	4 (7.5)	0.761
Initial septic shock	30 (18.0)	32 (60.4)	< 0.001
Pitt bacteremia score, median (IQR)	1(0–2)	4 (1.0–5.5)	< 0.001
Community-onset	94 (56.3)	31 (58.5)	0.778
Duration of bacteremia, median (IQR)	1 (1–5)	1 (1.0–3.5)	0.143
Vancomycin MIC (μg/mL)			0.483
≤0.5	57 (34.1)	15 (28.3)	
1	102 (61.1)	37 (69.8)	
2	8 (4.8)	1 (1.9)	
Teicoplanin MIC (μg/mL)			0.344
≤0.5	129 (77.2)	43 (81.1)	
1	13 (7.8)	6 (11.3)	
2	19 (11.4)	3 (5.7)	
4	5 (3.0)	0 (0)	
8	1 (0.6)	1 (1.9)	
Focus of infection			
Infective endocarditis	4 (2.4)	1 (1.9)	> 0.999
Osteoarticular infection	55 (32.9)	1 (1.9)	< 0.001
Pneumonia	18 (10.8)	26 (49.1)	< 0.001
Surgical site infection	9 (5.4)	0 (0.0)	0.118
Skin and soft tissue infection	8 (4.8)	1 (1.9)	0.691
Intravascular catheter	32 (19.2)	6 (11.3)	0.188
Unknown focus	29 (17.4)	16 (30.2)	0.044
Removal of eradicable focus			
Removal of focus after 3 d	18 (10.8)	3 (5.7)	0.269
Removal of focus before 3 d	52 (31.1)	3 (5.7)	< 0.001
Metastatic infection	16 (9.6)	6 (11.3)	0.713

Data are presented as numbers (%) unless otherwise indicated

CKD, chronic kidney disease; CVC, central venous catheter; IQR, interquartile range; MIC, minimum inhibitory concentration

**Table 2:** Characteristics of initial glycopeptide therapy in patients with methicillin-resistant *S. aureus* bacteremia

Characteristics	Survived (*n* = 167)	Deceased (*n* = 53)	*P* value
Initial vancomycin therapy	118 (70.7)	21 (53.8)	0.044
Initial trough concentration within 3 d (μg/mL), median (IQR), (*n* = 124)	10.8 (6.8–16.9)	12.0 (9.3–15.5)	0.912
Highest trough concentration within 7 d (μg/mL), median (IQR), (*n* = 130)	15.4 (10.0–20.2)	14.4 (10.6–19.0)	0.997
Initial teicoplanin therapy	49 (29.3)	18 (46.2)	0.044
Loading dose (mg/kg), median (IQR), (*n* = 62)	6.0 (5.6–6.7)	6.0 (5.5–6.8)	0.877
Maintenance dose (mg/kg), median (IQR), (*n* = 67)	6.0 (5.4–6.2)	6.0 (5.5–6.9)	0.557
Duration of Initial glycopeptide therapy, days, median (IQR), (*n* = 206 ^*a*^)	17 (12–29)	5 (3–13)	< 0.001
Time to glycopeptide therapy			
Interval from blood culture collection, hours, median (IQR), (*n* = 206 ^*a*^)	38 (21–73)	34 (5–55)	0.087
Within 3 h (*n* = 220)	15 (9.0)	7 (13.2)	0.372
Within 6 h (*n* = 219)	23 (13.8)	10 (19.2)	0.337
Within 12 h (*n* = 218)	29 (17.4)	12 (23.5)	0.324
Within 24 h (*n* = 216)	53 (31.7)	18 (36.7)	0.513
Within 48 h (*n* = 211)	100 (60.2)	23 (51.1)	0.271
Within 72 h (*n* = 201)	125 (75.3)	25 (71.4)	0.632

Data are presented as numbers (%) unless otherwise indicated

IQR, interquartile range

^a^ Patients who died before glycopeptide antibiotic administration were excluded

## Independent predictors of 30-day in-hospital mortality

All variables with *p* < 0.10 in the univariable analysis, along with clinically relevant variables, including sex, and community-onset, were entered into a multivariable logistic regression model (Supplementary Table 3, Additional File 1). The final model comprised 5 variables (Table 3), of which metastatic solid tumor, initial septic shock, pneumonia, and unknown focus were independently associated with 30-day in-hospital mortality. The goodness-of-fit of the final model was supported by a non-significant Hosmer–Lemeshow test (*p* = 0.133), indicating adequate calibration. The model also demonstrated excellent discrimination, with an AUROC of 0.879.

**Table 3:** Multivariable analysis of 30-day in-hospital mortality in patients with methicillin-resistant *S. aureus* bacteremia

Model ^*a*^	Variable	Adjusted OR	95% CI	*P* value
Total patients	Metastatic solid tumor	6.894	2.114–22.477	0.001
(*n* = 220, death = 53)	Initial septic shock	3.518	1.428–8.667	0.006
	Pneumonia	10.040	3.396–29.688	< 0.001
	Unknown focus	3.729	1.203–11.560	0.023
	Initial vancomycin therapy^*b*^	0.766	0.320–1.836	0.550

CI, confidence interval; OR, odds ratio

^*a*^ Hosmer-Lemeshow test: *p* = 0.133; area under the receiver operating characteristic curve = 0.879

^*b*^ Initial antibiotic therapy was classified into “initial teicoplanin,” “initial vancomycin,” or “no glycopeptide antibiotics,” and “initial teicoplanin” was used as the reference group for the analysis

## Effect of time to initiation of glycopeptide therapy on 30-day in-hospital mortality

**Fig. 3:**
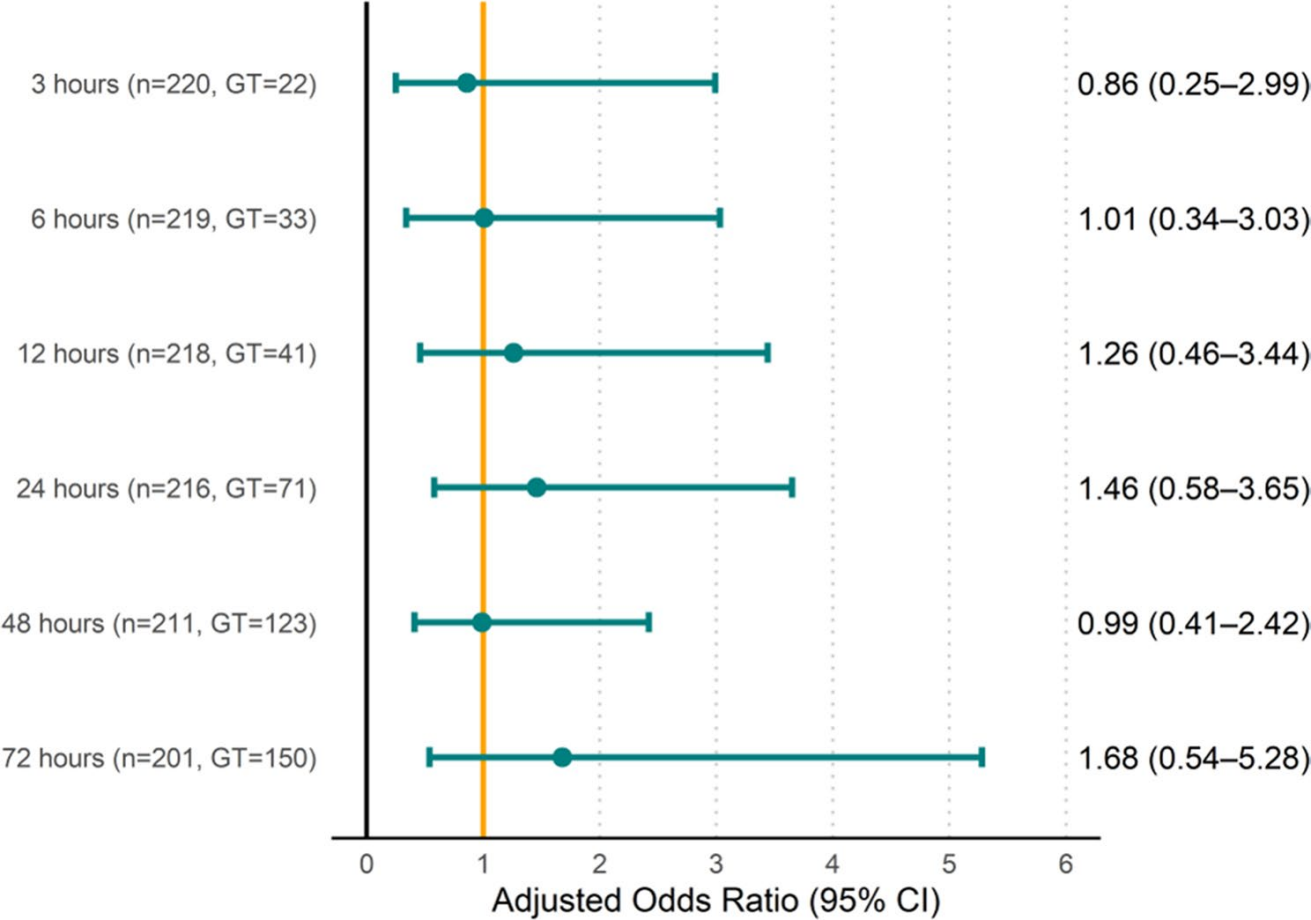
Adjusted odds ratios for 30-day in-hospital mortality by time to initial glycopeptide therapy in MRSA bacteremia. Adjusted odds ratios (aOrs) are shown relative to the group that did not receive glycopeptide therapy. ci, confidence interval; gt, glycopeptide therapy; MRSA, methicillin-resistant *S. aureus*

After adjusting for five covariates (Table 3), the time to initiate glycopeptide therapy was not significantly associated with 30-day in-hospital mortality at any predefined landmark (Fig. 3) (Supplementary Table 4, Additional File 1). Sensitivity analysis, which excluded patients with glycopeptide therapy initiation of > 120 h after the index blood culture, yielded similar results (Supplementary Fig. 1, Additional File 2) (Supplementary Table 5, Additional File 1).

## Discussion

In this study, we evaluated whether the time from index blood culture to the initiation of glycopeptide therapy affected 30-day in-hospital mortality in patients with MRSA bacteremia. After correcting for the immortal-time bias, we found no significant association between early glycopeptide initiation and 30-day mortality rates. Instead, host factors, such as metastatic solid tumor, along with host–pathogen interaction factors, such as initial septic shock, pneumonia, and unknown focus, emerged as significant predictors of mortality, which is consistent with previous studies [3, 17, 18]. In addition, we observed that the majority of deaths occurred within the first two weeks after the initial positive blood culture, a finding similar to previous reports [24].

Previous studies evaluating the effects of time to AAT in MRSA bacteremia have reported inconsistent results (Supplementary Table 6, Additional File 1) [4–14]. These discrepancies likely reflect differences in patient populations and methodological approaches, which require careful consideration. First, several MRSA bacteremia studies that reported no significant benefit of early AAT did not account for the immortal-time bias [4, 5, 8, 10]. Such bias can attenuate the effectiveness of early therapy because patients who survive longer inherently remain at risk of receiving delayed AAT [15]. Second, because the survival benefit of early AAT is most evident in patients with septic shock or organ dysfunction [11, 15, 16], cohorts that include a larger proportion of such patients are more likely to show a marked survival benefit from early therapy. This study used a landmark analysis to adjust for immortal-time bias and was performed in a cohort with a relatively high incidence of septic shock (28.2%). Nevertheless, we found no significant association between time to AAT and 30-day mortality, and a sensitivity analysis that excluded patients who received glycopeptide therapy > 120 h after the index blood culture produced similar results.

Additionally, the distribution of infection foci in SAB may have attenuated the effect of time to AAT because both mortality and incidence rates of metastatic complications vary substantially by focus [3, 25, 26]. In the majority of previous studies on MRSA bacteremia that failed to demonstrate a significant benefit of early AAT, catheter-related bloodstream infections (CRBSIs) accounted for a relatively large proportion of cases (Supplementary Table 6, Additional File 1) [4, 5, 10] and were associated with particularly low risk of mortality [3, 25]. This study included a relatively high proportion of osteoarticular infections (25.5%) compared with previous studies. Although osteoarticular infections are traditionally classified as intermediate-risk sources of SAB, together with soft tissue infections and unknown foci [3], osteomyelitis and prosthetic joint infections often follow a subacute course that lasts several weeks [27]. Furthermore, because osteoarticular infections often require source control [28], non-antibiotic interventions, combined with their typically slow clinical progression, may have blunted the impact of time to AAT on outcomes.

To control other antibiotic-related factors, we limited the initial antibiotic therapy to glycopeptides and evaluated the administered dosage. At both hospitals, vancomycin dosing was adjusted by TDM, and the median trough concentration exceeded 10 µg/mL. In contrast, teicoplanin was administered as weight-based doses without TDM, according to the clinician’s judgment. Consequently, teicoplanin was most often initiated at a 6 mg/kg loading dose, and in some cases, the loading dose was omitted, which may have resulted in suboptimal drug exposure in serious or complicated MRSA infections [29]. Given that teicoplanin therapy, compared with vancomycin, showed a trend toward higher mortality and a shorter time to AAT in the univariable analysis, underdosing of teicoplanin could have attenuated the benefit of early AAT.

This study had several limitations. First, because this study relied on retrospectively extracted electronic medical records, residual confounding by indication may have persisted. Although we adjusted for key covariates, including the presence of septic shock, patients with severe illness were more likely to receive broad-spectrum agents such as glycopeptides earlier, potentially attenuating the benefits of early AAT. Second, although the cohort spans five years of MRSA bacteremia across two university-affiliated centers, the absolute number of deaths was modest, resulting in wide confidence intervals around several effect estimates. Consequently, the study may have lacked sufficient power to detect clinically meaningful but minimal benefit of early AAT. Third, because this study, similar to most previous investigations, defined the baseline as the time of blood culture collection, the interval from symptom onset to culture acquisition is likely to be longer in community-onset infections than in nosocomial cases [4–10, 12, 13]. Moreover, this unmeasured pre-hospital delay may vary considerably across studies owing to differences in healthcare accessibility among countries and regions. Thus, variations in the proportion of community-onset infections, together with regional differences in healthcare accessibility, may have influenced the observed effects of early AAT. Fourth, as this study was conducted in only two hospitals within a single country, the generalizability of our findings to other healthcare settings or countries may be limited. Fifth, we did not perform a separate sensitivity analysis restricted to patients with septic shock. The limited number of events in this subgroup would have constrained the number of covariates that could be included and increased the risk of overfitting, potentially resulting in unstable or misleading estimates. Sixth, although vancomycin TDM was changed from trough concentration–based dosing to AUC–based dosing during the study period, trough concentration was used as the monitoring parameter for analysis to ensure consistency across all patients. In addition, the timing of TDM varied among patients, making it difficult to consistently determine the exact time to reach therapeutic levels. Seventh, this study included only patients with clinical signs or symptoms of infection, and therefore those with MRSA bacteremia without overt manifestations may have been excluded.

## Conclusions

Despite these limitations, our findings suggest that in patients with MRSA bacteremia, the time of glycopeptide initiation may exert a lower impact on clinical outcomes than comorbidities, overall severity of illness, or infection focus. Moreover, the clinical impact of time to AAT may differ according to the infection focus in MRSA bacteremia. Future large-scale studies that define the optimal time to AAT according to disease severity or infection focus could inform personalized antibiotic stewardship.

## Electronic supplementary material

Below is the link to the electronic supplementary material.

Supplementary Material 1

Supplementary Material 2

## Data Availability

The datasets used and/or analysed during the current study are available from the corresponding author on reasonable request.

## Abbreviations

AAT: Appropriate antibiotic therapy
AOR: Adjusted odds ratio
AUC: Area under the curve
AUROC: Area under the receiver operating characteristic curve
CCI: Charlson Comorbidity Index
CI: Confidence interval
CRBSI: Catheter-related bloodstream infection
IQR: Interquartile range
IRB: Institutional Review Board
MRSA: Methicillin-resistant *Staphylococcus aureus*
PBS: Pitt bacteremia score
SAB: *Staphylococcus aureus* bacteremia
TDM: Therapeutic drug monitoring
