# Supplementary material for: Time to initial glycopeptide therapy and 30-day mortality in methicillin-resistant Staphylococcus aureus bacteremia: a retrospective cohort study
Source: BMC Infect Dis. 2025 Nov 19;25:1614. doi: 10.1186/s12879-025-12040-9 (PMC12628965; doi:10.1186/s12879-025-12040-9)
Supplement: Supplementary file 1 — Supplementary Material 1 [file 12879_2025_12040_MOESM1_ESM.docx]

Supplementary Table 1 Baseline characteristics of patients with methicillin-resistant *S. aureus* bacteremia by hospital

| Characteristics | BPH (n = 122) | HPH (n = 98) | *P* value |
| --- | --- | --- | --- |
| Male | 66 (54.1) | 50 (51.0) | 0.649 |
| Age in years, median (IQR) | 71 (60.0–79.3) | 71.5 (62.0–80.3) | 0.283 |
| Comorbidities |  |  |  |
| Charlson comorbidity index, median (IQR) | 5 (3.0–7.3) | 5 (4–7) | 0.442 |
| Moderate to severe liver disease | 5 (4.1) | 5 (5.1) | 0.755 |
| Moderate to severe CKD | 23 (18.9) | 16 (16.3) | 0.626 |
| Serum creatinine (mg/dL), median (IQR) | 1.05 (0.66–1.84) | 1.20 (0.75–2.03) | 0.250 |
| Hemodialysis dependence | 15 (12.3) | 11 (11.2) | 0.807 |
| Metastatic solid tumor | 17 (13.9) | 9 (9.2) | 0.302 |
| Prostheses |  |  |  |
| Orthopedic device | 30 (24.6) | 17 (17.3) | 0.193 |
| Cardiovascular device | 6 (4.9) | 5 (5.1) | > 0.999 |
| Long-term CVC | 20 (16.4) | 19 (19.4) | 0.563 |
| Other prosthesis | 8 (6.6) | 7 (7.1) | 0.864 |
| Initial septic shock | 33 (27.0) | 29 (29.6) | 0.677 |
| Pitt bacteremia score, median (IQR) | 1 (0.0–3.3) | 2 (0.0–3.3) | 0.314 |
| Community-onset | 59 (48.4) | 66 (67.3) | 0.005 |
| Duration of bacteremia, median (IQR) | 1 (1–5) | 1 (1–4) | 0.219 |
| Vancomycin MIC (μg/mL) |  |  | < 0.001 |
| ≤0.5 | 26 (21.3) | 46 (46.9) |  |
| 1 | 92 (75.4) | 47 (48.0) |  |
| 2 | 4 (3.3) | 5 (5.1) |  |
| Teicoplanin MIC (μg/mL) |  |  | 0.079 |
| ≤0.5 | 93 (76.2) | 79 (80.6) |  |
| 1 | 13 (10.7) | 6 (6.1) |  |
| 2 | 11 (9.0) | 11 (11.2) |  |
| 4 | 5 (4.1) | 0 (0.0) |  |
| 8 | 0 (0.0) | 2 (2.0) |  |
| Focus of infection |  |  |  |
| Infective endocarditis | 3 (2.5) | 2 (2.0) | > 0.999 |
| Osteoarticular infection | 30 (24.6) | 26 (26.5) | 0.743 |
| Pneumonia | 19 (15.6) | 25 (25.5) | 0.067 |
| Surgical site infection | 6 (4.9) | 3 (3.1) | 0.734 |
| Skin and soft tissue infection | 6 (4.9) | 3 (3.1) | 0.734 |
| Intravascular catheter | 20 (16.4) | 18 (18.4) | 0.700 |
| Unknown focus | 32 (26.2) | 13 (13.3) | 0.018 |
| Removal of eradicable focus |  |  |  |
| Removal of focus after 3 d | 5 (4.1) | 16 (16.3) | 0.002 |
| Removal of focus before 3 d | 26 (21.3) | 29 (29.6) | 0.159 |
| Metastatic infection | 12 (9.8) | 10 (10.2) | 0.928 |
| 30-day in-hospital mortality | 29 (23.8) | 24 (24.5) | 0.901 |

Data are presented as numbers (%) unless otherwise indicated.

BPH, Busan Paik Hospital; CKD, chronic kidney disease; CVC, central venous catheter; HPH, Haeundae Paik Hospital; IQR, interquartile range; MIC, minimum inhibitory concentration.

Supplementary Table 2 Characteristics of initial glycopeptide therapy in patients with methicillin-resistant *S. aureus* bacteremia by hospital

| Characteristics | BPH (n = 122) | HPH (n = 98) | *P* value |
| --- | --- | --- | --- |
| Initial vancomycin therapy | 63 (52.9) | 76 (87.4) | < 0.001 |
| Initial trough concentration within 3 d (μg/mL), median (IQR), (n = 124) | 10.6 (8.5–15.8) | 11.9 (6.6–17.8) | 0.570 |
| Highest trough concentration within 7 d (μg/mL), median (IQR), (n = 130) | 14.0 (9.8–18.5) | 15.9 (10.7–21.5) | 0.142 |
| Initial teicoplanin therapy | 56 (47.1) | 11 (11.2) | < 0.001 |
| Loading dose (mg/kg), median (IQR), (n = 62) | 6.0 (5.6–6.5) | 6.0 (5.1–7.7) | 0.944 |
| Maintenance dose (mg/kg), median (IQR), (n = 67) | 6.0 (5.6–6.5) | 6.0 (4.6–6.6) | 0.300 |
| Duration of Initial glycopeptide therapy, days, median (IQR), (n = 206 *^a^*) | 18 (13–34) | 16 (7–27) | 0.021 |
| Time to glycopeptide therapy |  |  |  |
| Interval from blood culture collection, hours, median (IQR), (n = 206 *^a^*) | 31 (15–58) | 48 (24–76) | 0.002 |
| Within 3 h (n = 220) | 16 (13.1) | 6 (6.1) | 0.086 |
| Within 6 h (n = 219) | 22 (18.0) | 11 (11.3) | 0.169 |
| Within 12 h (n = 218) | 27 (22.3) | 14 (14.4) | 0.139 |
| Within 24 h (n = 216) | 49 (40.8) | 22 (22.9) | 0.005 |
| Within 48 h (n = 211) | 80 (68.4) | 43 (45.7) | 0.001 |
| Within 72 h (n = 201) | 93 (82.3) | 57 (64.8) | 0.005 |

Data are presented as numbers (%) unless otherwise indicated.

BPH, Busan Paik Hospital; HPH, Haeundae Paik Hospital; IQR, interquartile range.

*^a^* Patients who died before antibiotic administration were excluded.

Supplementary Table 3 Univariate analysis of 30-day in-hospital mortality in patients with methicillin-resistant *S. aureus* bacteremia

| Variable *^a^* | OR | 95% CI | *P* value |
| --- | --- | --- | --- |
| Male | 1.111 | 0.598–2.066 | 0.739 |
| Age (per 1 year) | 1.045 | 1.017–1.075 | 0.002 |
| Moderate to severe liver disease | 3.375 | 0.938–12.148 | 0.063 |
| Metastatic solid tumor | 2.654 | 1.135–6.208 | 0.024 |
| Initial septic shock | 6.959 | 3.534–13.702 | <0.001 |
| PBS (per 1 point) | 1.394 | 1.220–1.591 | <0.001 |
| Community-onset | 1.094 | 0.585–2.047 | 0.778 |
| Osteoarticular focus | 0.039 | 0.005–0.291 | 0.002 |
| Pneumonia | 7.971 | 3.851–16.499 | <0.001 |
| Unknown focus | 2.058 | 1.012–4.186 | 0.046 |
| Removal of focus before 3 days | 0.133 | 0.040–0.445 | 0.001 |
| Initial vancomycin therapy *^b^* | 0.484 | 0.238–0.988 | 0.046 |

CI, confidence interval; PBS, Pitt bacteremia score; OR, odds ratio.

*^a^* Variance inflation factors (VIFs) for variables were as follows: male, 1.065; age (per 1 year), 1.091; moderate to severe liver disease, 1.055; metastatic solid tumor, 1.095; initial septic shock, 2.343; PBS (per 1 point), 2.514; community-onset, 1.132; osteoarticular focus, 1.625; pneumonia, 1.990; unknown focus, 1.741; removal of focus before 3 days, 1.363; and initial vancomycin therapy, 1.077.

*^b^* Initial antibiotic therapy was classified into “initial teicoplanin,” “initial vancomycin,” or “no antibiotics,” and “initial teicoplanin” was used as the reference group for the analysis.

Supplementary Table 4 Effect of time to glycopeptide therapy on 30‑day in‑hospital mortality among patients with methicillin-resistant *S. aureus* bacteremia, evaluated at pre-defined landmark times

| Landmark time | GT/Patients | Adjusted OR (95% CI) | *P* value |
| --- | --- | --- | --- |
| Total patients (n = 220) |  |  |  |
| 3 h | 22/220 | 0.857 (0.245–2.994) | 0.808 |
| 6 h | 33/219 | 1.009 (0.336–3.028) | 0.987 |
| 12 h | 41/218 | 1.259 (0.460–3.442) | 0.654 |
| 24 h | 71/216 | 1.460 (0.584–3.647) | 0.418 |
| 48 h | 123/211 | 0.992 (0.407–2.417) | 0.986 |
| 72 h | 150/201 | 1.682 (0.536–5.275) | 0.373 |

GT, glycopeptide therapy; CI, confidence interval; OR, odds ratio.

Supplementary Table 5 Effect of time to glycopeptide therapy on 30‑day in‑hospital mortality among patients with methicillin-resistant *S. aureus* bacteremia, evaluated at pre-defined landmark times (excluding patients who received antibiotics after 120 h)

| Landmark time | GT/Patients | Adjusted OR (95% CI) | *P* value |
| --- | --- | --- | --- |
| Total patients (n = 208) |  |  |  |
| 3 h | 22/208 | 0.880 (0.247–3.128) | 0.843 |
| 6 h | 33/207 | 1.030 (0.338–3.137) | 0.959 |
| 12 h | 41/206 | 1.294 (0.465–3.601) | 0.621 |
| 24 h | 71/204 | 1.559 (0.610–3.984) | 0.354 |
| 48 h | 123/199 | 1.077 (0.421–2.754) | 0.877 |
| 72 h | 150/189 | 2.416 (0.611–9.559) | 0.209 |

GT, glycopeptide therapy; CI, confidence interval; OR, odds ratio.

Supplementary Table 6 Summary of studies evaluating the impact of time to appropriate antibiotic therapy in MRSA bacteremia

| Time to AAT–mortality association | Author, design, time period | Region | SAB/MRSA (%) | CAO/shock (%) | Focus/source control (%) | AAT time window/patients (%) | Outcome (%) | Adjusted effect of delayed AAT |
| --- | --- | --- | --- | --- | --- | --- | --- | --- |
| NS | Kim S-H, et al., retrospective cohort, case-control, 1998–2001 [4] | South Korea | 127/100 | 6/NA | Unknown (35.4%), CRI (27.5%), pneumonia (13.3%)/NA | 48 h from the onset/23.6 | SAB-related mortality (37.0) | aOR 1.1 (95% CI 0.4–3.1) |
|  | Fang C-T, et al., retrospective cohort,  1997–2001 [5] | Taiwan | 162/100 | 0/13.5 | CRI (44.4%),  SSTI (22.2%), unknown (19.1%)/NA | Per-day from blood culture/NA | 30-day mortality (36.4) | aHR 0.87 (95% CI 0.74–1.02) |
|  | Marchaim D, et al., retrospective case-control, 2001–2005 [8] | US | 399/100 | NA/NA (severe sepsis 26.2%) | NA/50.3% | <2 days from blood culture/24.5 | In-hospital mortality (42.2) | aOR 1.85 (95% CI 0.94–3.64); but significant in age ≥65 yr |
|  | Yoon Y, et al.,  prospective observational, 2010–2011 [10] | South Korea | 345/100 | HCAO 14.8/ 13.0 | CRI (51.3%), pneumonia (11.6%)/NA | 48 h from the blood culture sampling/44.6 | In-hospital mortality (33.0) | aOR NA |
|  | Retrospective cohort, 2018–2023 | South Korea | 220/100 | 56.8/ 28.2 | OA (25.5%), unknown (20.4%), pneumonia (20.0%)/34.5% | Pre-defined time-points (3, 6, 12, 24, 48, and 72 h)/  55.9 within 48 h | 30-day in-hospital mortality (24.1) | NS |
| Significant | Lodise TP, et al., retrospective cohort, 1999–2001 [14] | US | 167/61.7 | 0/NA | NA/NA | 44.75 h from the time of the first positive blood culture result/71.2 | Infection-related mortality | aOR 3.8 (95% CI 1.3–11.0) |
|  | Schramm GE, et al., retrospective cohort, 2002–2004 [6] | US | 474/100 | 6.3*/ 40.8* | NA/NA | 24 h after MRSA-positivity of a sterile-site culture/30.7* | In-hospital mortality (23.1*) | aOR 1.92 (95% CI 1.48–2.5) |
|  | Rodríguez-Baño J, et al., prospective cohort, 2003 [7] | Spain | 209/100 | HCAO 30.6/ 5.2 | SSTI (42.6%), unknown or CRI (15.8%),  pneumonia (10.5%)/NA | 48 h from blood culture/21.1 | 30-day mortality (23) | aOR 3.0 (95% CI 1.01–9.0) |
|  | Paul M, et al.,  retrospective cohort, 1988–1994 [9] | Israel | 510/100 | 0/ 12.7 | Unknown (24.1%), SSTI (14.5%),  CRI (10.7%)  /NA | 48 h from blood culture/32.9 | 30-day mortality (43.9) | aOR 2.15 (95% CI 1.34–3.46) |
|  | Corl KA, et al.,  retrospective cohort,  2003–2015 [11] | US | 506/52.9 | 100/ 100 | Pneumonia (44.5%), SSTI (26.9%),  UTI (24.9%)/NA | Per-hour delay from ED arrival/NA | 30-day mortality (78.1*) | aOR 1.11 (95% CI 1.02–1.21) |
|  | Kadri S, et al., retrospective cohort,  2005–2014 [12] | US | 6566/40.9 | 91*/ 14.5* | NA/NA | The day of blood culture sample/81* | In-hospital mortality (11%*) | aOR 1.85 (95% CI 1.43–2.40) |
|  | Ohnuma T, et al., retrospective cross-sectional [13] | US | 4470/100 | 95*/ 32.6* | NA/NA | The day or the day after the blood culture sample/ 97* | In-hospital mortality (14.9%*) | aOR 0.46 (95% CI 0.30–0.71), based on AAT |

* Data pertain to the overall population including the MRSA subset.

AAT, appropriate antibiotic therapy; aOR, adjusted odds ratio; aHR, adjusted hazard ratio; CAO, community-acquired onset; CI, confidence interval; CRI, catheter-related infection; ED, emergency department; HCOA, healthcare-associated onset; IAT, inappropriate antibiotic therapy; MRSA, methicillin-resistant *S. aureus*; NA, not available; NS, not significant; OA, osteoarticular infection; SSTI, skin and soft tissue infection; UTI, urinary tract infection.
