# Supplementary material for: Time to initial glycopeptide therapy and 30-day mortality in methicillin-resistant Staphylococcus aureus bacteremia: a retrospective cohort study
Source: BMC Infect Dis. 2025 Nov 19;25:1614. doi: 10.1186/s12879-025-12040-9 (PMC12628965; doi:10.1186/s12879-025-12040-9)
Supplement: Supplementary file 2 — Supplementary Material 2 [file 12879_2025_12040_MOESM2_ESM.zip › 12879_2025_12040_MOESM2_ESM/12879_2025_12040_MOESM2_ESM.pptx]

## Slide 1
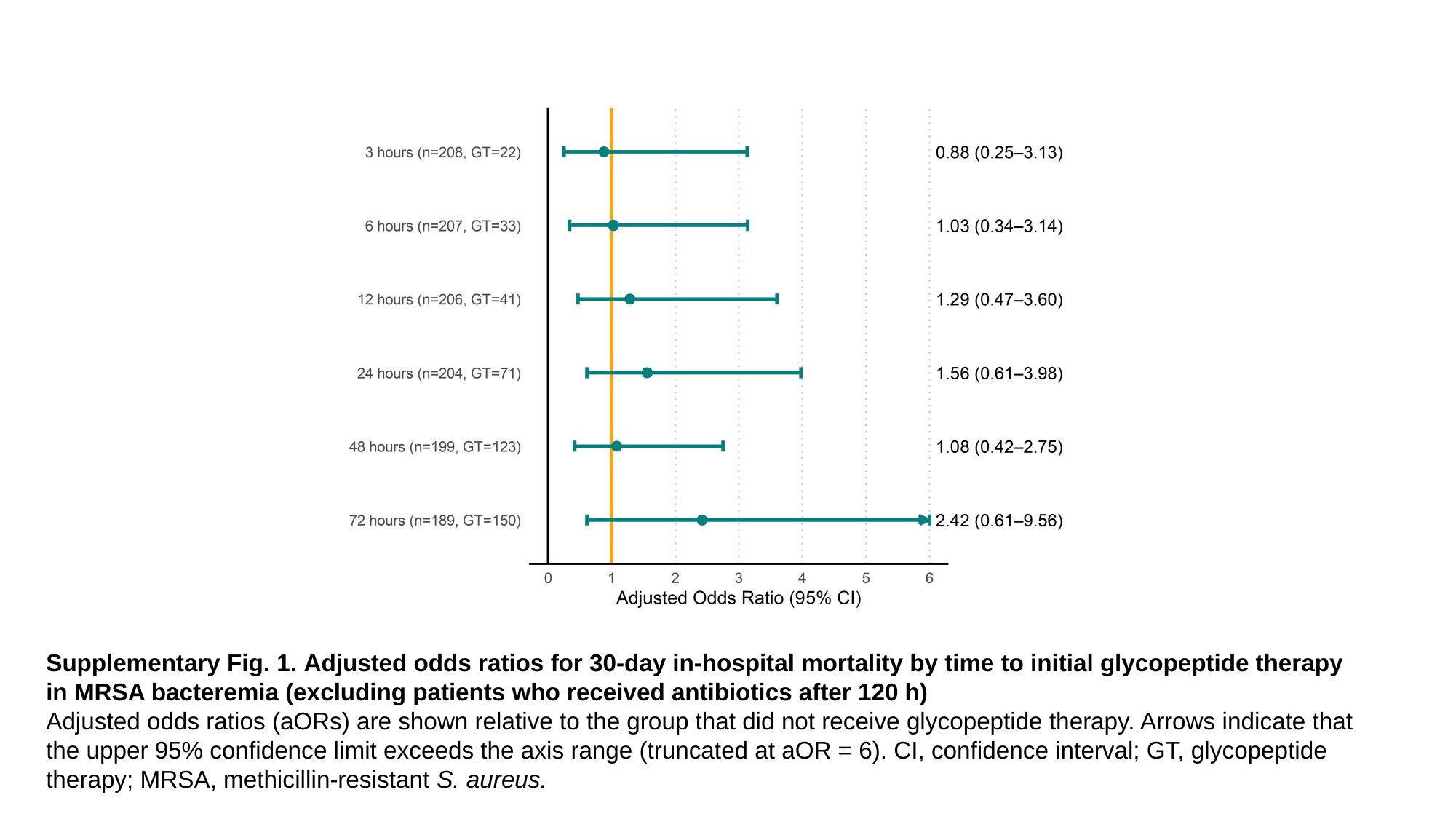

Supplementary Fig. 1. Adjusted odds ratios for 30-day in-hospital mortality by time to initial glycopeptide therapy in MRSA bacteremia (excluding patients who received antibiotics after 120 h)
Adjusted odds ratios (aORs) are shown relative to the group that did not receive glycopeptide therapy. Arrows indicate that the upper 95% confidence limit exceeds the axis range (truncated at aOR = 6). CI, confidence interval; GT, glycopeptide therapy; MRSA, methicillin-resistant S. aureus.
